# Three-Dimensional Shape Measurements of Specular Objects Using Phase-Measuring Deflectometry

**DOI:** 10.3390/s17122835

**Published:** 2017-12-07

**Authors:** Zonghua Zhang, Yuemin Wang, Shujun Huang, Yue Liu, Caixia Chang, Feng Gao, Xiangqian Jiang

**Affiliations:** 1 School of Mechanical Engineering, Hebei University of Technology, Tianjin 300130, China; 15022336051@163.com (Y.W.); hbhsj824@163.com (S.H.); ccaixia223@163.com (C.C.); 2 Centre for Precision Technologies, University of Huddersfield, Huddersfield, HD1 3DH, UK; Yue.Liu@hud.ac.uk (Y.L.); f.gao@hud.ac.uk (F.G.); x.jiang@hud.ac.uk (X.J.)

**Keywords:** phase-measuring deflectometry, phase calculation, slope integration, 3D calibration, 3D shape measurement, specular objects

## Abstract

The fast development in the fields of integrated circuits, photovoltaics, the automobile industry, advanced manufacturing, and astronomy have led to the importance and necessity of quickly and accurately obtaining three-dimensional (3D) shape data of specular surfaces for quality control and function evaluation. Owing to the advantages of a large dynamic range, non-contact operation, full-field and fast acquisition, high accuracy, and automatic data processing, phase-measuring deflectometry (PMD, also called fringe reflection profilometry) has been widely studied and applied in many fields. Phase information coded in the reflected fringe patterns relates to the local slope and height of the measured specular objects. The 3D shape is obtained by integrating the local gradient data or directly calculating the depth data from the phase information. We present a review of the relevant techniques regarding classical PMD. The improved PMD technique is then used to measure specular objects having discontinuous and/or isolated surfaces. Some influential factors on the measured results are presented. The challenges and future research directions are discussed to further advance PMD techniques. Finally, the application fields of PMD are briefly introduced.

## 1. Introduction

The three-dimensional (3D) shape measurement of objects is becoming increasingly important in many application fields. To obtain the 3D shape of diffuse objects, many optical measurement methods have been developed and the relevant techniques have been reviewed [1,2,3,4,5,6,7,8,9]. Many object surfaces exhibit special properties, such as being specular and transparent. For measuring transparent objects, a review paper has summarized the recent progress [10]. However, measuring the 3D shape of specular objects remains a challenge because of the reflecting property of their surfaces. Normally, contact and non-contact optical methods have been widely studied for measuring specular objects. The coordinate measurement machine (CMM) is a popular choice in contact methods [11]. Because a probe needs to touch the measured surface and scan along the 2D (two-dimensional) directions, CMM has the drawbacks of low speed, high cost, and potential scratching of the surface. Non-contact optical methods mainly include three classes for specular surface measurements: common structured light projection, interferometry and deflectometry.

To measure specular objects like diffuse objects, the surface properties need to be changed. In most cases, a powder spray is used to cover the measured specular object surfaces [12]. Then the sprayed surfaces can be measured using common structured light projection techniques, for example, full-field fringe pattern projection profilometry [13]. However, the coating will slightly change the geometry of the measured specular objects. Moreover, some objects, such as antiques and highly accurate optical components in aircrafts and automobiles, should not have powder sprayed onto their surfaces [14,15]. Therefore, interferometry and deflectometry have recently been used to study specular objects because these two methods do not require contact or changing the surface.

Interferometry uses the phenomenon of interference to obtain distance information. It is a superior technique for measuring simple surfaces such as spheres, planar surfaces, and weakly aspheric surfaces. A reference is normally required for interferometry; therefore, it is hard to measure complicated aspheric mirrors or free-form specular objects. White light interferometry [16], wavelength scanning interferometer [17] and multiple wavelength interferometer [18] can be used to measure specular objects having discontinuous surfaces. However, the field of view of the objective lens, the range of the scanner and the limited synthetic wavelength are restricted to the measurement range. These modes of interferometry are normally used for surface finish measurements instead of surface form measurements. Therefore, interferometry is not suitable for measuring a greatly curved and/or large scale surface, although it can measure specular surfaces with a very high accuracy and resolution.

To measure free-form specular objects with a steep slope and/or large size, in recent years, deflectometry has been investigated by many researchers, which has led to the development of different deflectometric techniques, such as Moire deflectometry [19,20,21,22], the Ronchi method [23,24], phase-measuring deflectometry (PMD, also called fringe reflection profilometry) using structured illumination of the surface [25,26,27,28,29,30], and laser scanning deflectometric techniques [30,31,32,33,34,35]. Compared with interferometry, deflectometric techniques do not need to locate the specular objects under investigation at a precise position. Although these techniques overcome the shortcomings of interferometry to measure specular objects with a high dynamic range, it remains a challenge to reconstruct a specular surface shape by using deflectometric methods. Deflectometry calculates the slope (surface gradient) distribution of the surface, and finally the 3D shape is usually obtained by numerically integrating the slope distribution. Because the phase can yield continuous and highly accurate data, PMD-based methods have been widely studied for measuring specular objects. Other benefits of PMD are a large dynamic range, non-contact operation, full-field measurement, and automatic data processing. Therefore, this paper focuses on reviewing the recent developments and the existing problems of PMD.

Most of the existing PMD-based methods have problems when measuring the specular objects having multiple discontinuous and/or isolated surfaces. Many improved PMD methods have been studied to overcome the challenges in classical PMD. This paper will also briefly introduce these improved PMD methods and their recent development, especially two new PMD methods. One is called the DPMD (direct PMD) method which uses two liquid crystal display (LCD) screens plus a beam splitter to realize the parallel design of the two screens. The other is MPMD (model PMD) which involves a mathematical model to simultaneously reconstruct both the height and the slopes from the measured phases, instead of integrating just the slopes.

The following section will review the relevant techniques in classical PMD, mainly including the generation of fringe patterns, the geometric calibration, and the slope data integration. Section 3 introduces some improved PMD methods to measure the 3D shape of specular objects having discontinuous and/or isolated surfaces. The influential factors on the measurement results are given in Section 4. Section 5 discusses the challenges and the future research directions for the advancement of the PMD techniques. Some application fields of PMD are briefly reviewed in Section 6. The conclusive remarks are provided in Section 7.

## 2. Principle

**Figure 1:**
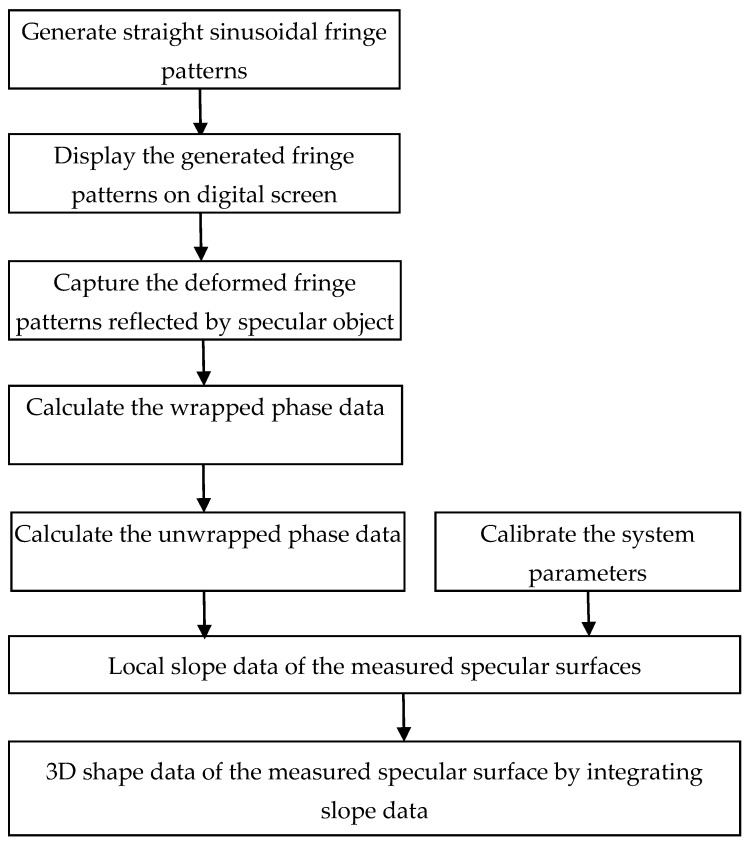
Flowchart of 3D shape measurement of specular objects by using classical PMD.

The classical PMD technique uses the full-field fringe patterns to measure the slope information and then slope integration to obtain the 3D shape data of the specular objects. Digital sinusoidal fringe patterns are commonly generated using computer software. The generated fringe patterns are displayed on a digital screen, such as an LCD, or projected onto a ground glass plate. From a different viewpoint, the reflected fringe patterns from the specular surfaces appear deformed with regard to the slope variation of the measured surfaces and the modulated fringe patterns are captured by an imaging device, for example, a charged couple device (CCD) camera. In PMD systems, one, two and multi-cameras can be used. However, few researchers studied PMD by using multi-cameras because the reflected ray at one point by the specular surface is along a certain direction according to the law of reflection. The reflected ray can be only captured theoretically from one certain direction. Therefore, most of the existing PMD systems contain one or two cameras. A stereo-camera PMD system can simplify the geometric calibration comparing to using mono camera. Wrapped phase information in the captured fringe patterns is demodulated by using multiple-step phase-shifting algorithms or transform-based algorithms. Spatial phase unwrapping and temporal phase unwrapping methods can be used to obtain the absolute phase data. Phase calculation and unwrapping methods are out of the scope of this paper and the readers should refer to the published literature for more details on these procedures [36,37,38,39,40,41,42,43,44]. The absolute phase data are applied to calculate the slope of the measured specular surfaces using the system parameters. Finally, the 3D shape of the specular surfaces under investigation is reconstructed by integrating the slope data. The procedure of the classical PMD is illustrated in Figure 1. The following subsections will elaborate on each step in the classical PMD.

### 2.1. Fringe Reflection

**Figure 2:**
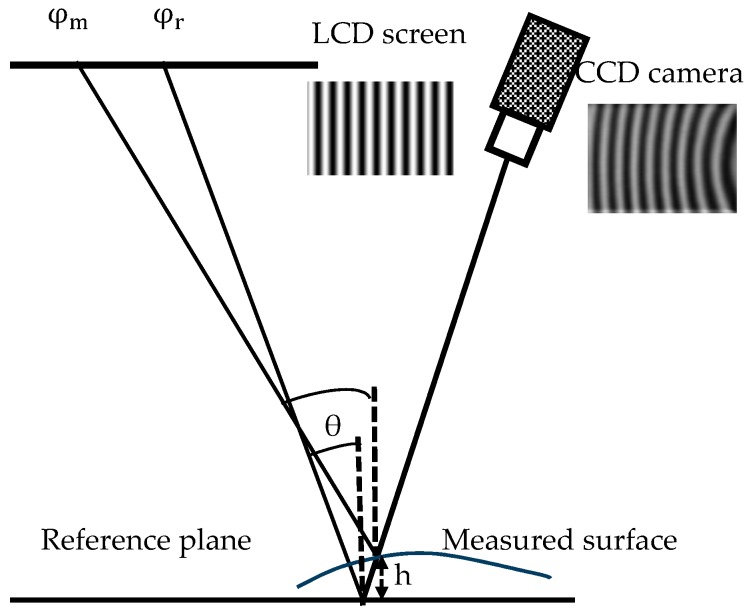
Principle of classical PMD to measure specular objects.

The most commonly used full-field optical reflection profilometry methods are the analysis of the reflected structured light patterns from the measured specular objects, as illustrated in Figure 2.

The two orthogonal local slopes data need to be integrated to reconstruct the 3D global shape. Therefore, vertical and horizontal fringe patterns usually need to be displayed sequentially on an LCD screen and captured by an imaging device. As the imaging device will take a longer time to finish capturing the sequential fringe patterns, it is therefore difficult to measure the specular object surfaces in real-time or in a dynamic way. To reduce the acquisition time, cross fringe pattern and color fringe pattern methods have been studied to code multiple fringe patterns in one image.

#### 2.1.1. Orthogonal Fringe

**Figure 3:**
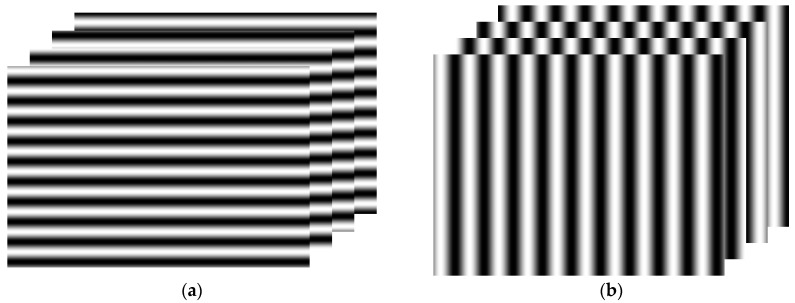
Orthogonal fringe pattern sets. (**a**) Horizontal direction and (**b**) vertical direction.

Most researchers have used the sequential displaying of two sets of orthogonal fringe patterns on the LCD screen to calculate orthogonal phase information [25,45,46]. Horizontal and vertical fringe patterns can be generated by a computer and then sequentially displayed on the screen, as shown in Figure 3. These fringe patterns are reflected by the surface of the measured specular objects. The reflected fringe patterns are deformed and captured by an imaging device, such as a CCD camera. The deformed fringe patterns contain the phase information, which correspond to the slope and 3D shape of the surface of the measured specular object. The captured fringe patterns are saved for post processing.

#### 2.1.2. Crossed Fringe

**Figure 4:**
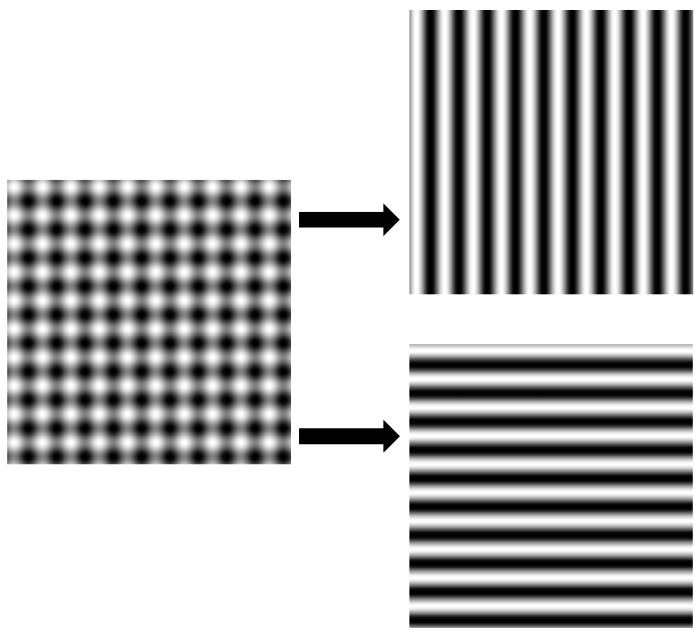
One crossed fringe pattern containing two orthogonal fringe patterns.

To avoid sequentially capturing fringe patterns in two directions, cross fringe patterns are used to simultaneously display orthogonal fringe patterns to reduce the acquisition time, as illustrated in Figure 4. Two fringe patterns (one vertical and one horizontal) can be extracted from one crossed fringe pattern. If Fourier fringe analysis is used, vertical and horizontal wrapped phase data can be calculated from the two extracted fringe patterns. Two orthogonal unwrapped phase maps can be obtained by using a spatial phase unwrapping algorithm from a single-shot image. Therefore, it is possible to measure the 3D shape of a dynamic specular surface by using one crossed fringe pattern in PMD methods.

Huang et al. proposed a method for measuring the dynamic 3D shape for a specular surface with a monoscopic fringe reflection technique and the 2D WFR (windowed Fourier ridge) algorithm [47]. Only one cross grating was needed to reconstruct the 3D surface shape changes. The dynamic 3D shape of a continuous specular surface of a water wave has been investigated to prove the feasibility of the proposed method. With an optimized Fourier evaluation, high quality data of the smooth optical surfaces could be acquired with a single shot by using the cross fringe patterns [48]. The authors also demonstrated a novel phase-shift technique by using the cross fringe patterns to implement a 1D translation of the fringe pattern, instead of the common 2D translation achieved in PMD. Therefore, a 1D N-phase shift allowed for the acquisition of the two orthogonal phases, with only N exposures instead of 2N exposures. Xie et al. proposed the orthogonal composite fringe reflection technique to measure specular surfaces [49]. In this technique, the phase distributions in two directions were extracted by a 2D Fourier algorithm from the distorted composite fringe pattern. However, there was a non-ignorable spectrum overlapping phenomenon of the frequency spectrum in the two directions when the tested surface was free-form and complex, and this resulted in a phase error when a 2D Fourier algorithm was used to extract the phase from this composite fringe pattern. In another study [50], the authors proposed a fast temporal phase unwrapping algorithm to extract the two integer phases for each frequency instead of two groups of phase shift fringes by using the orthogonal grid fringes for the fringe reflection technique. The ridge errors in the direct unwrapped phases were significantly suppressed by a pseudo-phase-shift strategy without any extra captured fringes. The fringe amount used for unwrapping was greatly reduced by this proposed method. Xu et al. proposed a deflectometry method by using the quaternary orthogonal grid fringes to retrieve the surface slopes [51]. Because only one image was required to extract the two perpendicular direction phases, the proposed method was robust against environmental noise and vibrations. The color of each pixel was encoded by the red, green and blue components, so that two perpendicular fringe patterns were composed of quaternary orthogonal grid fringes. Their proposed method could measure the dynamic change of specular objects. Liu et al. proposed an improved PMD for wavefront measurements using a composite fringe to reduce the projection fringes and improve the accuracy [52]. The single composite fringe contained four fringes in different directions. Two high frequency orthogonal fringe patterns and two single period orthogonal fringe patterns could be obtained from the composite fringe by a fast Fourier transform, which could be used to obtain the unwrapped phase by a heterodyne method. Finally, the 3D shape of specular objects was reconstructed by the integration of the derivatives. Therefore, the proposed method was more applicable to dynamic wavefront measurements by using only one composite fringe image. Flores et al. proposed a 2D PMD method that used a 2D additive fringe to extend the PMD from 1D to 2D [53]. The deformed fringe patterns in two orthogonal directions were acquired in a single-shot frame. Two wrapped phase maps were calculated by the Fourier transform method. Therefore, the potential application of the proposed PMD method is the characterization of dynamic phase objects that require high acquisition rates. However, phase unwrapping must be carried out before shape information can be deduced from the partial derivatives of the phase. For an object having a complex shape, the phase unwrapping will become a difficult procedure.

#### 2.1.3. Color Fringe

Owing to the rapid development of imaging devices, especially the emergence of color 3CCD cameras, which contain three-chips [54], it is possible to develop a more rapid fringe encoding and decoding process. In this situation, color-encoded fringe patterns have been widely studied and used as carrier channels for measuring diffuse surfaces in fringe projection profilometry to reduce the required number of captured fringe images [55,56,57,58]. Color-encoded fringe projection techniques are used to measure dynamic objects.

**Figure 5:**
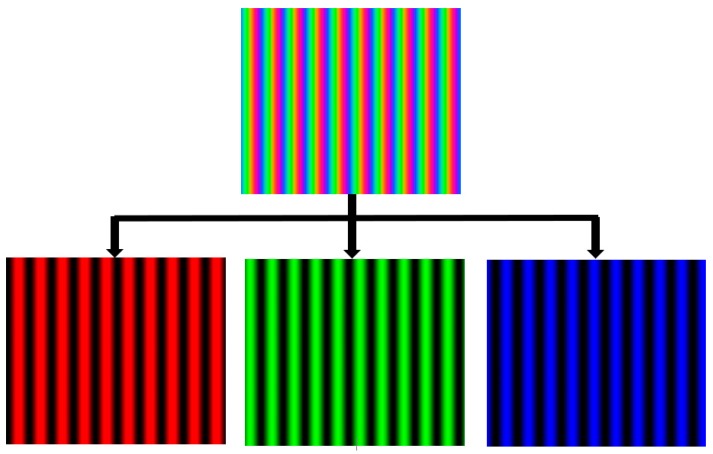
One color composite fringe pattern containing three fringe patterns.

This kind of technique can be adopted in PMD since the commonly used LCD screen has three major color channels like the color CCD camera. In fact, some researchers have studied color fringe pattern reflection techniques by using the main red, green and blue channels as carriers. Fringe patterns having a shifted phase of −2π/3, 0 and 2π/3 are coded into the major red, green and blue channels to generate a color fringe image, as shown in the top image illustrated in Figure 5. After capturing the reflected color fringe pattern of a specular object surface, three fringe patterns can be extracted from the red, green and blue channels for post phase calculation, as shown in the bottom three fringe patterns in Figure 5. Therefore, the wrapped phase data can be calculated from one captured image by using a three-step phase shifting algorithm.

If crossed fringe patterns are coded into the three color channels of a color image, six fringe patterns (including three vertical phase shifted fringe patterns and three horizontal phase shifted fringe patterns) are extracted from one color image. Therefore, two orthogonal wrapped phase maps and then vertical and horizontal local slope data can be obtained from a single-shot image. Based on this technique, an instantaneous phase shifting deflectometry method was presented previously [59]. In this paper, the authors presented an instantaneous phase shifting deflectometry measurement method by multiplexing phase shifted fringe patterns with color, and decomposing them in x and y using the Fourier technique. A time varying deformable mirror has been measured by the instantaneous method. However, the 3D shape data were reconstructed by integrating the slope data.

Wu et al. presented a color-encoded fringe reflection technique to measure a dynamic specular surface [60,61]. Because the horizontal and vertical fringe patterns were coded into the red and blue channels respectively, only one color-encoded fringe pattern was required to measure the slope data with a high accuracy and then the 3D shape information was obtained by integrating the slope data along two orthogonal directions. An experiment with a wafer proved the capability of the proposed color-encoded fringe reflection method to measure the dynamic specular surface. Xu et al. proposed a new deflectometry method by using the quaternary orthogonal grid fringes to retrieve the surface slopes [51]. Because only one image was required to extract the two perpendicular directional phases, the proposed method was robust against environmental noise and vibrations. The color of each pixel was encoded by the red, green and blue components, so that two perpendicular fringe patterns composed the quaternary orthogonal grid fringes. The proposed method could measure a dynamic change of specular objects. A phase retrieval algorithm based on color-frequency encoding was proposed to obtain absolute phase maps [62]. The red and blue channels of a color 3CCD camera were used to code dual-frequency fringe patterns. The crosstalk between color channels could be ignored because there were no overlapping spectra between the red and blue channels.

In summary, the three fringe reflection methods have their merits and disadvantages, as shown in Table 1. One crossed fringe pattern contains two fringe patterns, so it can reduce the acquisition time a half of orthogonal fringe reflection. However, resolution and accuracy of the calculated phase will decrease because the intensity of each pixel overlaps two values of two fringe patterns. Because three color channels are simultaneously used to code fringe patterns, color fringe reflection can reduce the acquisition time 1/3rd of gray fringe reflection. However, crosstalk and chromatic aberration between color channels will greatly affect the measured results.

### 2.2. Phase Extraction

After capturing the deformed fringe patterns reflected by the measured specular object surfaces, phase information can be calculated. The captured fringe patterns, whether gray or color, have the same properties as those for measuring diffuse objects. Therefore, the existing fringe analysis algorithms can be used to calculate the wrapped phase and the unwrapped phase. Multiple-step phase-shifting algorithms and transform-based algorithms (including Fourier transform, windowed Fourier transform, and wavelet transform) are used to calculate the wrapped phase data. The unwrapped phase data can be obtained by using spatial phase unwrapping methods and temporal phase unwrapping methods. The wrapped and unwrapped phase calculation methods are outside of the scope of this paper and interested readers can refer to the published literature [36,37,38,39,40,41,42,43,44].

### 2.3. Geometric Calibration

PMD only measures the local slope of specular surfaces. To obtain the global 3D shape, the slope data need to be integrated, which is a procedure that is sensitive to systematic errors. Therefore, it is necessary and important to accurately calibrate the system parameters in PMD, which builds up the optical ray reflection geometry in a common coordinate system among optical devices, such as an LCD screen, a CCD camera and a mirror, by using absolute phase tracing. Calibration is an essential topic in optical metrology. The calibration procedure of PMD includes camera calibration and geometric calibration. The camera calibration determines the internal parameters, including two focal lengths, two principal point coordinates and four image radial and tangential distortion coefficients. There have been many studies concerning the camera calibration [63,64] and a popular calibration method has been proposed by using a checkerboard plane proposed by Zhang [65]. Therefore, camera calibration is a mature research field.

The geometric calibration procedure of PMD involves localizing the position of the camera, the screen and the object used in the investigation in the same coordinate system. However, this procedure is complicated and difficult, because the screen is out of the field of view of the camera. A planar mirror is widely used to reflect the screen, so that the camera can directly observe and capture the virtual image of the reflected screen. The existing geometric calibration methods can be classified into three categories: a plane mirror with markers [25,45], a plane mirror [66,67,68,69,70], and an auxiliary device or moving device [71,72].

The authors have presented a method to calibrate the poses between the camera and screen by introducing a reference plane mirror with fiducials that is precisely aligned at the same place as the test surface and in the field of view of the camera [25,45]. Moreover, the physical positions of the fiducials are pre-located by an additional precision measurement system such as the stereo vision-based photogrammetric method.

To solve this problem, some authors used a planar mirror without markers for more than three times to implement the camera calibration and to determine the geometrical relationship between the screen and the camera with the concept of bundle adjustment [66,67]. By placing the mirror in three different positions in front of the camera, the poses of the screen with respect to the camera could be estimated [68]. Furthermore, the reflection ray direction corresponding to each camera pixel was determined during the camera calibration and the orthogonal iteration algorithm was introduced into the pose estimation for global optimization. Therefore, the proposed method has the ability to flexibly achieve a high accuracy and strong anti-noise capability. Olesch et al. proposed a self-calibration method to deal with the inaccurate data and to reduce the global accuracy [69]. They expanded the calibration method to arbitrary specular surfaces. The proposed self-calibration method was an iteration of three different steps. Finally, the global accuracy reduced to below 1 μm for a planar mirror on an 80 mm × 80 mm field. E et al. presented a method to measure an optically flat surface with a high accuracy [70]. This method required that the last orientation of the checker pattern was aligned at the same position as the test optical flat. A reference optical flat with a high quality surface was measured to further reduce the influence of the calibration errors. After subtracting the figure of the reference flat from the figure of the test flat, the system errors could be eliminated.

Xiao et al. proposed a geometrical calibration method by using a pose transfer between the screen and camera [71]. An auxiliary camera and an auxiliary LCD screen were introduced into the proposed calibration method. The calibration results mainly depended on the accuracy of the pose estimation of the screens and cameras. Pose estimation for a calibrated camera was the problem for determining the orientation and position of the camera with respect to the object coordinate frame. Therefore, the proposed calibration method was not affected by the property of a flat mirror with and/or without markers on the surface. Yuan et al. proposed a geometric calibration method to move the LCD screen once and then found the points on the LCD with different positions that corresponded to the same camera pixels by using correspondence matching [72]. The proposed method could easily calibrate the reflection ray directions of the camera without using an external stop.

### 2.4. Slope Integration

The classical PMD methods just measure the local slope information of the specular object surface, instead of the global 3D shape. To obtain 3D shape data from the slope data, many kinds of 2D integration methods have been studied. However, every integration method is very sensitive to the systematic errors and an initial value is required to obtain accurate 3D shape information owing to the height-slope ambiguity. For a continuous specular surface, one known surface point is sufficient to solve the ambiguity. Starting from this point with the known normal, the height information of all other points can be calculated by numerical integration. However, it is difficult to obtain the position of even one single point on a specular surface. To solve this problem, many integration methods have been studied.

Three methods have been proposed to obtain the incident light vector: (1) A telecentric setup of the measuring system. An imaging lens is used to image the screen at infinity. Therefore, each point defines only one direction of the incident light. The drawback of this method is the required size of the imaging lens and thus the dynamic range of the measuring system. (2) Two measurements for different screen positions. The display screen is located at two different positions to get an additional point in space. However, mechanically translating the screen during the measurement has the disadvantages of being laborious, time consuming and inaccurate. (3) Stereo vision. Two calibrated cameras capture the reflected fringe patterns simultaneously. According to the calculated normal on one point from the two cameras should be identical, the height information for the given point is obtained.

In general, slope integration can be classified as the following categories: the RBF (radial basis function)-based method, the least-squares method, and the transform-based method. In 2015, Huang et al. compared the three types of 2D integration methods for shape reconstruction from gradient data under various conditions for both reconstruction accuracy and processing speed [73]. The merits and disadvantages of each integration method were mainly presented to show their different performance in accuracy and speed, so that one could properly choose a suitable integration method for a particular application. In this paper, the recent developments of the three gradient integration methods are mainly reviewed.

#### 2.4.1. RBF-Based Method

The RBF-based methods that applied RBFs into the PMD technique were first proposed by Ettl et al. [74]. An interpolation function tailored for gradient data was expressed with a weighted combination of analytical derivatives of the selected RBFs. By matching the analytical derivatives with the measured slope data values, the shape data could be obtained in a least squares sense. Owing to its relatively large memory consumption, the RBF-based method generally divides the slope dataset into smaller subsets and then stitches the resultant data after slope integration on each subset because the RBF is mainly effective at handling small size measurement data owing to the computational intensity of using RBFs for large data sets. Therefore, for a practical measurement, the RBF generally divides the gradient dataset into a set of overlapping image subsets and then merges them together using the overlapping regions.

Ren et al. presented a novel least-squares integration method, which employed the RBF integration method as a supplementary constraint to reconstruct the surface shapes with nonrectangular samples [75]. To improve the integration process from gradient to shape, Huang et al. proposed a framework with a combination of the RBFs method and the least-squares integration method [76]. The advantages of the proposed method were accuracy, automatic, easily implemented, and robust for the stitching of the datasets.

#### 2.4.2. Least-Squares Method

The least-squares method assumes that the model shape is a biquadratic spline in the Southwell configuration. However, the surface in practical measurements may not be described by a biquadratic function. To solve this problem, many researchers studied different methods to improve the least-squares integration method.

Li et al. proposed an optimized reconstruction method by using high-order numerical differentiation formats [77]. Guang et al. presented a higher-order iterative finite-difference-based least-squares integration (HIFLI) method to improve the accuracy and convergence rate of shape reconstruction [78]. The HIFLI method is a hybrid of the iterative finite-difference-based least-squares integration (IFLI) method and a higher-order gradient reconstruction method. Therefore, the proposed integration method inherits the advantages of the IFLI and HIFLI methods. Ren et al. proposed a modified easy-implementation integration method called EI-FLI based on finite-difference-based least-squares integration [79], which could work in arbitrary domains, and could directly and conveniently handle incomplete gradient data. To carry out the proposed algorithm in a practical stereo deflectometry measurement, gradients were calculated in both CCD frames, and then were mixed together as original data to be meshed into a rectangular grid format. Simulations and experiments showed that this modified method was feasible and could work efficiently. Later, the same authors proposed an improved least-squares integration method to integrate non-uniform gradient data [75]. The proposed method was based on the assumption that a regular surface at a given point could be approximated using Taylor’s theorem by a polynomial. Therefore, within a tiny region around this point, the normal vector of one point was perpendicular to the vectors connecting points at either side. The proposed method effectively handled the gradient data with non-uniform grids. To improve the accuracy of the integration process from gradient to shape and to solve the issue of the incorrect biquadratic shape assumption, Huang et al. proposed an iterative compensation technique to the traditional least-squares integration method [80]. The proposed method was accurate, fast, and able to handle large data sets, and so was an effective and accurate 2D integration tool for handling shape from slope problems in some gradient measuring-based optical inspection applications.

Xiao et al. presented a sparse representation into PMD to enhance the defect detection ability [81]. A suitable integration method for PMD should preserve the local details of the measured surface without propagating the error along a certain path while reconstructing the global shape with high accuracy. There are always non-integrability conditions for the measured slope data, because of various kinds of image noises in the measured gradient field. Thus, scratches on the measured specular surface cannot be distinguished. To solve this problem, a sparse representation was used for refining a non-integral to an ideal integral field, whereas a standard least-squares method was used to achieve an ideal integral field. Huang et al. presented a novel integration method to handle 2D shape reconstruction from local slope data [82]. The proposed method employed splines to fit the measured slope data with piecewise polynomials. The analytical polynomial functions with pre-determined coefficients were employed to calculate the height variations in a lateral spacing by using the linear least squares method. Because of the high accuracy of spline fitting, the proposed method exhibited a high reconstruction accuracy and good performance at the boundaries of the datasets or holes. However, the proposed method only dealt with slope data in rectangular grids, and it exhibited a limitation for processing the slopes in quadrilateral grids and triangular grids.

The least-squares methods can be further divided into modal methods and zonal methods. The modal methods use a sum of relevant polynomials to approximate the tested surfaces and then obtain the coefficients by applying the least-squares method. However, the reconstructed results of the model methods are not precise when fitting complicated surfaces. The zonal methods are based on the relationship between height and slopes in a local zone. The unknown height value can be expressed by a matrix form and are solved by using matrix iterative methods. However, the convergence is slow and the stability is poor.

Li et al. proposed an improved zonal method to enhance the accuracy of the reconstructed 3D shape in PMD [83], by taking into account the unequally spaced or non-uniform sampling. The proposed method provided a high accuracy of the 3D shape data; however, it could extract the local detail information with a high frequency, such as a slight surface scratch on the test optical component. Huang et al. proposed a simple, straightforward, and efficient approach to deal with the zonal wavefront reconstruction in a quadrilateral geometry [84]. The proposed method could be regarded as a more general form of the zonal wavefront reconstruction. A new consideration on the height-slope relations was performed by taking the height increments as connectors instead of using the intermediate slopes. Therefore, an improvement could be realized by simple modifications from the existing zonal methods for a rectangular geometry. The presented method has the advantages of a higher accuracy, the ability to handle a large incomplete slope dataset in an arbitrary aperture, and the low computational complexity comparable with the classical zonal method.

To solve the drawbacks of the model and zonal methods, Zhou et al. presented a more stable and efficient method to reconstruct a 3D shape in PDM [85]. The proposed method used the coarse height information from a modal estimation as the initial iterative values. The real 3D shape values were accurately calculated by using a modified zonal reconstruction algorithm. The proposed method exhibited a high accuracy, rapid convergence and good stability.

#### 2.4.3. Transform-Based Method

The transform-based methods further include a discrete Fourier transform (DFT) or a discrete cosine transform (DCT). Freischlad et al. used DFT to reconstruct the wavefront [86]. However, the DFT algorithm operates in rectangular domains, it only provides a suboptimal solution with its implicit periodic boundary conditions and there are some missed pixels in the measured slope data. These will result in problems in the integration process. To solve these problems, many authors have proposed various improvement methods. The authors provided an iterative DFT method to extrapolate the missing slopes outside an arbitrarily-shaped aperture [87,88]. Poyneer et al. proposed a method to extrapolate the slope data with boundary and extension methods for a Hudgin or Fried configuration by taking into account the loop continuity [89]. The finite-difference solvers were used in the Gerchberg iteration to estimate the wavefront for general-shaped pupils in a Southwell configuration [90].

Although the DCT method can reduce the reconstruction error [91], it cannot be directly applied to incomplete gradient data for shape reconstruction. To solve this problem, the authors presented an iterative method for shape reconstruction from the gradient by using iterative DCT to deal with the integration problem without a complete gradient data set in a Southwell configuration [73], so that an arbitrarily-shaped aperture, or incomplete gradient data with missing slopes either inside a “hole” or outside the aperture could be solved by employing iterative DCT. The proposed method has the following advantages of the high accuracy of the DCT integration, the high accuracy of extrapolation outside an aperture with DCT-delivered derivatives through the Gerchberg iteration, and the high efficiency of the FFT algorithm.

Li et al. compared the transform-based method and the least-squares method [92]. Although the least-squares method provided better integrated results than the transform-based method, it took longer to process the gradient data than the latter.

#### 2.4.4. Comparison

The merits and disadvantages of the three integration methods are briefly compared for choosing the suitable integration method for measuring specular objects by using the classical PMD methods, as listed in Table 2.

## 3. Improved Phase-Measuring Deflectometry

The classical PMD-based methods need to integrate the slope data to reconstruct the absolute surface shape. However, the integration procedure is very error-prone because random errors will accumulate into large shape deviations, so that the measurement results will be inaccurate. Moreover, the classical PMD cannot directly measure complicated specular objects having discontinuous and/or isolated surfaces because of the procedure of slope integration.

The classical PMD techniques calculate the local slopes from the phases with pre-known height values and then integrate the slopes for the final height distribution with an accuracy of micrometer. Therefore, there is an inherent ambiguity in classical PMD regarding calculating the height and slopes of the specular surfaces under investigation. Owing to the uncertainty of the prior knowledge on the height, the slopes will be determined with errors and these errors will be propagated into the resultant height through the integration process. The height-slope ambiguity issue was partially solved by roughly assuming a prior height distribution [30], a height known seeding point [93], iterative reconstruction [69], using stereo vision [25], or the carrier removal method [94] to obtain the slope-related phase.

Based on the ray intersection, Petz et al. proposed a method of reflection grating photogrammetry (RGP) to measure discontinuous specular surfaces [45]. The tested points were independent of the others since this method employed ray intersection instead of integration. Xiao et al. presented a fringe reflection photogrammetry (FRP) method by importing a constraint bundle adjustment into RGP to reduce the effects of lens distortion on the measurement results [95]. Although the absolute coordinates of discontinuous specular surfaces can be obtained by these two methods, they do not provide a direct relationship between the height and phase information and require a spatial geometry computation. Moreover, the translated distance by a translating stage has great effects on the measured accuracy, especially when the axis of the translation and surface normal of the reference plane are not parallel.

Guo et al. presented a technique for measuring the 3D shapes of specular surfaces by using least-squares light tracking [28]. This technique involved moving the LCD monitor on the tracks to two or more different positions along the vertical direction. At each position, sinusoid fringe patterns were displayed on the LCD screen and reflected by the measured specular surface. From another viewpoint, the reflected fringe patterns were distorted and captured by a CCD camera for post data processing. Phase distributions at each position were measured and the locus of the incident ray for each pixel was determined in the least-squares sense from the phases, so that the 3D shape of the specular surface was reconstructed by tracing the incident ray. Although the proposed technique eliminated the computational complexities, phase ambiguities, and error accumulations, the accuracy of the moved tracks will have a great effect on the measurement results.

Tang et al. presented an advanced PMD method to measure an aspheric mirror [27]. Although the absolute height of the aspheric mirror having a large range of surface geometries could be measured, the measured accuracy depended on the accuracy of the moving components of the CCD camera and the LCD screen. The method could not measure the surface with a high deviation to paraboloid. The measuring procedure was complicated because the CCD camera was moved to several positions and the LCD screen to two positions, both along the optical axis of the tested mirror. However, in the actual situation, it was very difficult to realize this point. The used beam splitter caused aberration and limited the measurement size of the test surface. Later, the authors [96] improved on this method by locating the camera beside the optical axis of the test surface, so that the aspheric mirror with a high deviation could be measured. The improved method still requires moving the LCD screen to a known distance along the optical axis of the tested mirror. Moreover, the absolute height of the tested aspheric mirror was obtained by slope integration.

Zhao et al. presented a PMD method to test an aspherical mirror [97]. The proposed method required a reference screen to be located in two different positions from the mirror under investigation. Using the phase data, the original ray of every image point and its corresponding deflected ray could be constructed, so that their intersection points and the surface normal could be calculated. Through numerically integrating the surface normal, the aspheric mirror surface was reconstructed. Because the LCD screen was required to be moved to two different known positions by a linear translation stage during measurement, the measured accuracy was limited by the translation stage.

To further advance the development of PMD, two improved deflectometry methods of direct PMD (DPMD) [98,99,100,101] and model PMD (MPMD) [102,103,104] have been presented, which will be reviewed in the following two subsections, respectively.

### 3.1. Direct Phase-Measuring Deflectometry (DPMD)

**Figure 6:**
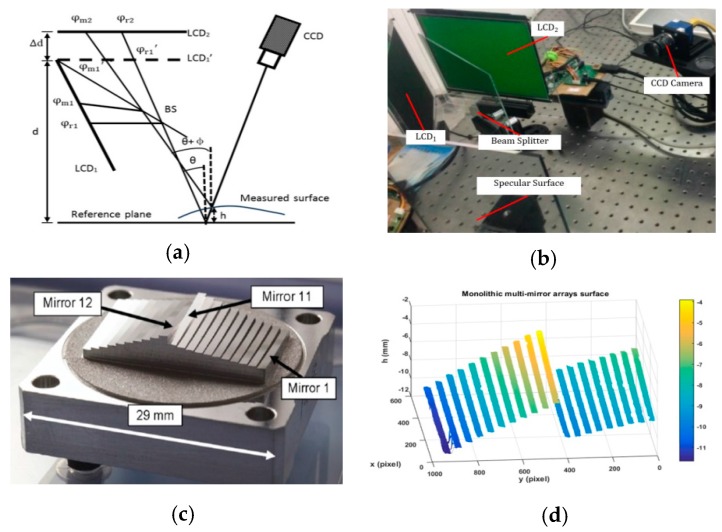
Measurement example of DPMD. (**a**) Principle of DPMD. (**b**) Hardware of the experimental setup. (**c**) Monolithic multi-mirror array on the MIRI spectrometer optics for the James Webb Space Telescope. (**d**) Measured depth.

A new direct PMD (DPMD) method has been developed to solve the problems of the existing classical PMD methods, as illustrated in Figure 6 [98,99,100]. DPMD builds a direct relationship between the phase and the depth, so that the 3D shape of the specular object is calculated directly from the phase data, instead of integrating the local slope data which are also obtained from the phase data. When an LCD screen is located at two known positions, a mathematical model is derived to directly relate the phase to the depth, instead of the slope of the tested specular surfaces. A plate BS (beam splitter) is used to realize the parallel design of two LCD screens and thus avoid the requirement to mechanically move a screen to two positions. Sinusoidal fringe patterns having the optimum fringe numbers are generated using software and are displayed on the two screens. A CCD camera captures the two sets of deformed fringe patterns. The wrapped and the absolute phase maps are calculated using a four-step phase-shifting algorithm and an optimum three-fringe number selection method, respectively. Because the optimum three-fringe number selection method calculates the absolute phase pixel by pixel, the depth information of the specular objects with discontinuous surfaces can be obtained directly from the calculated phase maps. Using the developed DPMD system, the authors successfully measured the 3D shape of a monolithic multi-mirror array on the MIRI spectrometer optics for the James Webb space telescope [105].

Therefore, the main advantages of DPMD compared with PDM are the following: (1) Error accumulation is avoided because DPMD does not need to integrate slope data. (2) Only if the phase data are obtained pixel by pixel, the 3D shape of specular objects having discontinuous and/or isolated surfaces can be directly measured from the absolute phase data.

Zhao et al. proposed a virtual measurement system to optimize the system parameters and evaluate the performance of the system for DPMD applications [101]. Four system parameters were analyzed to obtain accurate measurement results. The four system parameters included the distance d between screen LCD_1_ and the reference plane, the distance ∆*d* between screen LCD_1_ (the virtual image of LCD_1_) and screen LCD_2_, the angle *θ* between the optical axis of the camera and the reference plane, and the fringe period *P*. Researchers can therefore select suitable system parameters for actual DPMD (including PMD) measurement systems to obtain the 3D shapes of specular objects with an accuracy of sub-micrometer.

Comparing to a stereo PMD-based system, the DMPM system contains two parallel screens and one camera, instead of two cameras. Therefore, the depth information of a specular surface can be directly calculated from two absolute phase maps on the two screens.

### 3.2. Model Phase-Measuring Deflectometry (MPMD)

Huang et al. proposed a model PMD (MPMD) to represent the height and slopes of the specular surface using a mathematical model [102,103,104]. They introduced the modal wavefront reconstruction methods with a deeper consideration on simultaneously reconstructing both the height and the slopes from the measured phases, instead of just integrating the slopes. The model was updated by optimizing the model coefficients to minimize the discrepancy between the reprojection in the ray tracing and the actual measurement. In addition, the screen geometry from the calibration was used as the initial values and optimized using surface shape optimization. The proposed model can be applied to mono-PMD, stereo-PMD, and multi-cameras PMD configurations. The essential idea of MPMD is to represent both the height and slopes of the surface under investigation with well-established mathematical models and to optimize the coefficient vector to best explain the captured fringe patterns in the measurement. The coefficient vector and the position of the screen in camera coordinates are optimized by minimizing the differences between the reprojection and the measurement on the screen among the multiple pairs of correspondence in a least-squares sense. This nonlinear least-squares problem then can be solved by the Levenberg-Marquardt algorithm.

Recently, the authors analyzed the errors arising from the mismatch between the surface under investigation and the mathematical model being used [104]. To reduce the errors, a method was proposed to calculate the slope residuals and further reconstruct the local structures by using the zonal shape reconstruction.

## 4. Influential Factors on Measurement Results

To obtain accurate 3D shape data in the classical PMD methods and the improved PMD methods, it is necessary to analyze the influential factors on the measurement results. They mainly include the following parts: nonlinear response of display and imaging components, flatness and refraction of the display screen, slope integration, calibration and phase error.

### 4.1. Nonlinear Response of Display and Imaging Components

All the display and imaging devices possess a nonlinear response to adjust to human vision. However, a nonlinear response will change the standard shape of a sinusoidal fringe pattern into a non-sinusoidal shape, so that the calculated phase is incorrect [106,107,108]. Some researchers have used a binary fringe pattern to replace the sinusoidal fringe pattern on the side of the display screen [109]. By defocusing the imaging or projecting lens, the captured image contains a deformed sinusoidal fringe pattern. This technique decreases the contrast of the fringe pattern, so that the calculated phase is inaccurate and the obtained shape data is noisier. Another method is to compensate for nonlinear response by using a software-based technique [58]. For example, a look-up table (LUT) is built by projecting (displaying) different gray level intensities and capturing the corresponding gray value. The nonlinear response is corrected by generating a sinusoidal fringe pattern with the software.

### 4.2. Display Screen

In PMD, a pattern generator screen (for example, a TV screen or LCD screen) is required to be twice as large as the object under test. Therefore, the measurement of some specular objects requires a large screen. There are two main sources that affect the 3D shape measurement results: a flatness deviation of the display surface and refraction effects in the transparent layers of the display. The used screen to display fringe patterns is assumed as an ideal plane for phase calculations in PMD. In an actual situation, the manufactured screen is not flat. Especially, when the measured specular objects are large, it is very hard to meet this requirement [110]. The measurement results will have a systematic error if the flatness deviation is not compensated. To solve this problem, some researchers have studied different methods to calibrate the flatness deviation of the display screen.

In deflectometry, the commonly used LCD screen has refraction effects occurring in the transparent layers of the display, which means the observed pattern is not located at the surface of the screen. Typically, a display panel consists of a liquid crystal layer sandwiched between two glass substrates, and a number of other transparent layers. Therefore, the observed pattern is located below the display surface. Because the standard glass thickness of the transparent layers is in the range of 0.7 mm to 0.8 mm, the refraction effect results in an angle-dependent displacement of the observed pattern in the same order of magnitude. In fact, a linear model may be sufficient to significantly reduce the systematic errors induced by refraction.

Fischer et al. evaluated and analyzed LCD monitors for PMD application [111]. Their theoretical analysis and experiments showed that an in plane switching (IPS) technology developed by Hitachi in 1996 was more suitable for a PMD measurement system in terms of the observation-angle independent shape of the grayscale-characteristic curve.

The actual surface geometry of the LCD screen needs to be determined for a highly accurate measuring system. Software-based methods could be applied to compensate for the flatness deviation of a display screen. To calculate the 3D shape of the display screen, Petz et al. presented an object grating method [45]. This method combined pattern matching techniques with a photogrammetric evaluation by showing a regular grid pattern on the LCD display. An eighth-order polynomial fitting method was used to the measured data. However, the authors did not discuss how to compensate for the flatness deviation of the LCD screen. Ritter et al. presented a method to measure the deformation by using electronic speckle pattern interferometry and the object grating [112].

Maestro-Watson et al. proposed a method to calculate not only the index of refraction and the thickness of the refractive layer, but the non-flat shape of the LCD screen for PMD systems by regarding the LCD screen as a single glass layer model [113]. A coordinate measuring machine was employed to obtain the front surface shape of the LCD screen. The characterization of the front surface shape was described by a mathematical function. A checkerboard was placed on the front surface to determine its orientation in the camera coordinate system. Virtual markers were displayed on the screen and captured by a camera from different positions and orientations. The captured images showed view-dependent displacements of the displayed features owing to the refraction. Photogrammetric techniques were applied to relate the displacements observed in the images and the measured front surface, and a mathematical minimization algorithm was employed to estimate the parameters, including the thickness, the index of refraction and the flatness deviation of the LCD screen.

### 4.3. Slope Integration

During the procedure of slope integration, the random noise and systematic errors from the calculated gradient data will be accumulated to result in a big error of the reconstructed 3D shape data, as discussed previously. One solution is to explore a more robust 2D slope integration method. The other method is to avoid the procedure of slope integration, for example, the improved PMD methods of DPMD [98] and MPMD [102], so that the 3D shape data of specular objects can be directly calculated from phase information.

### 4.4. Calibration

Calibration is a procedure to determine the relationship between phase and slope (or shape) in deflectometry. Although many geometric calibration methods have been developed, the calibrated parameters have great effects on the final measurement results. Therefore, new calibration methods need to be developed to determine the accurate systematic parameters.

### 4.5. Phase Error

Since the slope data or shape data are calculated from the phase, an error in the phase has an effect on the accuracy of the defects and heights of the measured specular surfaces. Especially, the classical PMD will amplify the phase errors because the shape data are reconstructed by slope integration and the captured fringe pattern is defocused. Phase error sources mainly arise from the random noise, nonlinear response function, the non-telecentric imaging of the CCD camera, and the nonlinear response function of the LCD screen [114].

## 5. Future Directions

### 5.1. Invisible Light PMD

In PMD, the surface reflectivity is primarily influenced by the roughness of the measured surface. When the measured object surfaces are partially diffused, the reflected fringe patterns will have low quality because of light scattering. By using light of a longer wavelength, the specular reflection on surfaces can be obtained, while exhibiting a diffuse optical appearance in the visible light spectrum. However, for measuring a transparent object, the back side reflection is suppressed to avoid an incoherent superposition of signals originating from the front surface by using light of a shorter wavelength. Dynamic thermal patterns generation is an unsolved problem because the existing monitor or projector serves the purpose of a visible light spectrum. Therefore, displaying and imaging fringe patterns into a channel with a longer wavelength of the IR (infrared) and/or the UV (ultraviolet) spectrum is a research direction in PMD.

Höfer et al. applied IR DMP for the inspection of raw material surfaces in the steel or automobile industries [115]. Na et al. investigated the scattering effect on the measuring results at different wavelengths in PMD [116]. An appropriate wavelength of the light source could be chosen to reduce the scattering effect. Fine metal masks (FMMs) having partially specular surfaces have been measured by using near-IR deflectometry to obtain better reflected fringe patterns. Experiments on FMMs subjected to different stresses show a validation and effectiveness for measuring the variations in the shape of the surfaces. However, the shape data need to be integrated from the measured slope information. Kim et al. presented an IR deflectometry system to accurately measure local slopes and rapidly provide highly accurate surface profiles of freeform optical surfaces [117]. The system used a scanning thermal long-wave IR source to produce an IR spectrum that was reflected efficiently by rough optical surfaces.

Sprenger et al. developed UV deflectometry using an ultraviolet light source [118]. The back side reflex of a transparent object could be completely suppressed by using a UV light source instead of visible light. Therefore, each imaging pixel received a signal reflected by the front surface, without incoherent superposition from the back surface reflection of the transparent object. However, there were also no suitable spatial light modulators in the required wavelength range of the UV. Therefore, the authors introduced a method to move a slit mask by a linear motor across the virtual screen rather than a phase-shifted sinusoidal pattern.

### 5.2. Discontinuous and Isolated Specular Surfaces

Because most existing deflectometry methods require integrating slope data to obtain a 3D shape, specular objects having discontinuous and/or isolated surfaces cannot be measured directly. Although some researchers have studied techniques to solve this problem, many unsolved challenges remain. For example, an FRP method has been used to measure the absolute coordinates of discontinuous specular surfaces [95]. However, this method requires a complicated spatial geometry computation. Moreover, the distance translated by a translating stage strongly affects the measurement accuracy, especially when the axis of translation and surface normal of the reference plane are not parallel. Zhang et al. implemented a zonal wavefront reconstruction algorithm to realize a 3D, highly reflected and specular surface reconstruction [119]. Although gauge blocks with two different heights were tested successfully, it was difficult to measure multiple discontinuous and/or isolated surfaces because this method requires that each measured surface of the step object has one perturbed stripe.

### 5.3. High Accuracy

The measurement accuracy of the existing PMD methods can reach a level of micrometer, and even nanometer scale by using micro-objectives [26]. There are many factors that affect the accuracy of the measured 3D shape data in PMD, mainly the flatness deviation of the display screen, refraction effects of the display surface, non-linear response of displaying components and imaging device, calibration, phase calculation, and so on. In order to further improve the accuracy to reach sub-micrometer level, these errors need to be compensated. Most of these parts have been discussed in the Section “Influential factors on measurement results”.

### 5.4. Color Fringe

This is a new direction in deflectometry, especially in dynamic specular surface, online inspection, and real-time acquisition. Major color channels of the display and imaging devices can be chosen as carriers to code multiple fringe patterns simultaneously, so the acquisition time reduces to one third that of the gray fringe pattern when simultaneously using red, green and blue channels to code three fringe patterns. However, new problems arise in color fringe patterns, such as crosstalk and chromatic aberration between color channels, like that in color fringe projection profilometry [56,120].

### 5.5. Real-Time Acquisition (Dynamic Specular Surface Measurement)

There are many moving specular objects in actual situations, for example, on-line dynamic inspection [47,51,60,61]. For the fast or real-time acquisition of the 3D shape of the dynamic specular objects, some researchers used color fringe patterns to realize a single-shot measurement [51,60,61], while others used a Fourier transform to calculate phase information from one fringe pattern [47,53].

### 5.6. Portable

The existing PMD systems mainly consist of one or two LCD display screens with the size of a computer monitor, commercial CCD cameras, and a PC, which are heavy and inconvenient to assemble together, carry around and install in various environments. It is important to develop portable PMD systems to improve the accessibility of deflectometry. To realize this purpose, Huang et al. developed a compact 3D shape imaging system with the fringe reflection technique to measure small specular samples [121]. However, the system requires that the measured sample be almost placed at the same height level where the reference mirror was at, which is very hard to realize in actual situations. The 3D shape was then reconstructed from the gradient data. To design a lighter PMD system, Butel et al. presented a portable deflectometry technique running on an Android™ smartphone with a front-facing camera to quickly measure the slopes of eyeglasses, lenses, or mirrors within a minute [122]. The nonlinear response and automatic gain control of the smartphone were compensated for a highly accurate data acquisition. The experiments demonstrated that the presented technique could measure any specular 3D objects. Maldonado et al. developed a new portable deflectometry device, called slope-measuring portable optical test system (SPOTS), which achieved a 1 nm root mean square (RMS) surface accuracy for the mid-to-high spatial frequencies, and 300 nrad RMS slope precision [123]. The authors discussed the principles of operation, measurement modes, design, performance and error analysis of the proposed device. The portable PMD system exhibited a high spatial resolution, low slope uncertainty, and low measurement error. Röttinger et al. developed a miniaturized PMD system specifically for machine-integrated PMD measurements within an ultraprecision diamond turning machine [124]. The system was directly mounted onto the B-table of the lathe, allowing for a measurement of the workpieces without releasing them from the vacuum chuck.

### 5.7. Partial Reflective Surface

Some components have specular and non-specular parts on the same object surface. It is a challenging problem to measure the 3D shape of these kinds of objects. Skydan et al. provided a solution for measuring non-full-field reflective surfaces by using a masking algorithm and phase stepping profilometry [125]. To identify areas with no fringe patterns, a masking algorithm was applied to separate the valid and invalid data. The surface reconstruction procedure was demonstrated mathematically and algorithmically. Measurement of a spherical mirror with a known radius was performed to show the performance of the proposed technique. The proposed technique could partially solve the problem of measuring objects having areas such as holes on the surface or boundary shapes. However, for objects having multiple discontinuous surfaces, it was very hard to provide an effective masking area. Later, the authors applied the non-full-field reflective technique to measure automotive glass to speed up and ensure product development and manufacturing quality [126].

Zhang et al. presented a method to measure the surface contour by using a retroreflective grating analysis [127]. The grating was formed by attaching a grating to the retroreflective screen. The reflected grating will be bent and shifted owing to the variation of the slope and captured by a CCD camera for post-processing. A fast Fourier transform algorithm was used to extract the phase information. Slope data could be calculated from the phase data. The depth of each point on the surface of the specimen was obtained by integrating the slope and assuming a zero depth at the starting point. Later, the authors introduced a method of optically generating a grating by projection to flexibly adjust the properties of the grating to a specific application and/or to move the grating as a whole for phase shifting technology [128]. The waviness of a quantified artifact having a reflective surface has been tested by the proposed method.

### 5.8. Deflectometry Combining Interferometry

Although the classical and improved PMD methods can measure specular complex objects, such as an aspherical surface and free-form surface, it has low accuracy. Deflectometry is a gradient based surface profilometry, so it needs to integrate the measured data to obtain surface shape. However, interferometry is an accurate and well-known method for precision shape measurements. Some authors compared the merits and disadvantages of the two measuring methods for specular objects [129,130]. In fact, it is possible to combine deflectometry and interferometry together to measure large specular objects with a large dynamic range, and high accuracy and reliability. Hanayama presented a concept of combining the measurement by deflectometry for a global shape and interferometry for a local shape around a flat area [131]. Deflectometry can yield global and precise data with a large dynamic range, while interferometry measures a small area accurately and reliably.

## 6. Application Fields

### 6.1. Aspherical and Spherical Mirrors

Aspherical mirrors can provide the same function as spherical mirrors, but have small size and light weight. It is important to accurately inspect the 3D shape of the manufactured aspherical mirrors to ensure they satisfy the designed function. Lee et al. used the Ronchi test to measure the aspheric surfaces [23]. Guo et al. presented an improved PMD method to measure aspheric specular surfaces based on the geometric principle of the Ronchi test [46]. Xiao et al. presented an FRP method to obtain the 3D shape of aspherical mirrors by importing a constraint bundle adjustment into RGP to reduce the effects of lens distortion on the measurement results [95]. Tang et al. presented an advanced PMD method to measure the height of an aspherical mirror having a large range of surface geometries [27,96]. Later, the authors proposed a method based on fringe reflection to test the optical axis of an aspherical mirror with high accuracy [132]. Zhao et al. presented a PMD method to test the aspherical mirror by locating a reference screen at two different positions from the mirror under test [97]. Su et al. applied the developed PMD method called software configurable optical test system (SCOTS) to measure an X-ray mirror with a precision and accuracy better than 100 nrad (RMS) and ~200 nrad (RMS), respectively [133]. Fang et al. have reviewed current research on manufacturing and measuring freeform optics [134].

### 6.2. Automobile (Automotive Glass, Car Body)

The automobile industry is a popular research field in academia with substantial applications in daily life. There are many specular components that need to be inspected during vehicle manufacturing, such as the automotive side glass and car body. Skydan et al. applied the non-full-field reflective technique to measure automotive side glass to speed up and ensure product development and manufacturing quality [125,126]. Hoefling et al. presented a new method for measuring reflective objects by using phase information and applied it to detect shape defects on car body sheets [135]. The surface curvature and then the discrimination of defects were directly calculated from the phase data. Quantitative height information could also be derived from the surface curvature by using two integration steps. Xu et al. presented an integrated inspection system to simultaneously obtain the 3D shape, reflection normal and transmission distortion of the automotive glass based on analyzing the distorted fringe patterns [136].

### 6.3. Flaw Detection

The classical PMD methods and the improved PMD methods have been widely applied to detect defects, flaws and cracks on specular object surfaces. Hoefling et al. presented a new method for measuring and detecting shape defects on car body sheets by using phase information [135]. Hung et al. proposed a nondestructive subsurface flaw testing method by using reflected fringe patterns from specularly reflective objects [137]. The proposed method was simple, robust, and applicable in many industrial environments. Chan presented an optical reflective fringe pattern technique to detect subsurface cracks on objects having specular or semi-specular reflective surfaces [138]. The phase difference in the vicinity of a flaw corresponded to anomalies in the surface slope in the same vicinity of the flaw. Kammel et al. described a strategy to measure the curvature of the specular surfaces based on deflectometry [139]. The measured curvature was compared with the data of a reference object, so that the defects and/or a precise assessment of the specular surface quality could be performed.

### 6.4. Silicon Wafers and Ball Grid Array

Some small specular objects, for example, wafers and ball grid arrays (BGAs), require small fields of view for the imaging lens and display screen to measure the 3D shape information. Many researchers have studied the relevant techniques. Häusler et al. introduced a novel microdeflectometry technique to measure the microtopography of specular surfaces by using a micro-objective [26]. The lateral resolution was better than 1 micrometer and the height resolution was in the range of 1 nm. Krey et al. presented a fast optical scanning deflectometer to measure the topography of large silicon wafers [33]. Song et al. presented a novel structured light approach by encoding strip edges in the illuminated patterns to directly measure micro shiny targets, such as shiny coins, metallic workpieces, and BGA bumps [140].

### 6.5. Positioning of Specular Freeform Surface

It is important to precisely align optical freeform surfaces between the practical and model coordinates in the processes of their machining, measuring, and applications. However, it is a challenge to accurately position the optical freeform surfaces owing to their complex shapes and lack of references, which greatly constrains their effective applications. Zhang et al. proposed an easy-operation, low-cost and efficient method to accurately position optical freeform surfaces during machining based on PMD [141]. The basic concept, calculation model, and verification experiments were implemented in detail in their paper. The phase similarity and phase range of the fringes were used to obtain the position values with a high accuracy. This simple system based on PMD realized easy mounting for on-machine measurement. Some experiments were conducted and the results proved the effectiveness and high accuracy of the proposed method.

## 7. Conclusions

With the advent of intelligent manufacturing, there are many demands for measuring the 3D shape of specular objects to satisfy quality control and function requirements. As an emerging technique, PMD-based methods have been widely studied because of their advantages of a large dynamic range, non-contact operation, full-field and fast acquisition, high accuracy, and automatic data processing. Therefore, it is necessary to summarize the recent progress and new problems associated with PMD methods. This paper reviews the progress of PMD, including fringe pattern generation, phase calculation, geometric calibration, and slope integration to obtain 3D shape data for specular objects. Some improved PMD methods are discussed, especially DPMD and MPMD, for measuring specular objects having discontinuous and/or isolated surfaces. Then, the influential factors on the 3D measurement results are summarized. Finally, the paper also proposes the future research directions to advance PMD-based methods for actual application fields.

Although some improved PMD methods can measure specular objects having complicated surfaces, such as discontinuous and/or isolated components, there are many unsolved challenges that remain to measure their 3D shape accurately and quickly. Researchers need to improve the performance based on the measuring principle of deflectometry. However, an interesting direction is to combine different measuring techniques, for example deflectometry and interferometry. Deflectometry provides global shape information with a large dynamic range while interferometry measures local 3D data with high accuracy and precision.

## Figures and Tables

**Table 1:** Comparison of three fringe reflection methods.

	Orthogonal Fringe	Crossed Fringe	Color Fringe
Time	Long	Short	Shortest
Accuracy	High	Median	Low
Resolution	High	Median	High

**Table 2:** Comparison of three integration methods.

	RBF-Based	Least-Squares	Transform-Based
Speed	Slow	Fast for small size Very slow for huge size	Fast
Accuracy	High (sub-micrometer)	High (sub-micrometer)	Median (micrometer)
Memory	Huge for large size	Small	Large
